# An unusual case of spontaneous multi vessel coronary artery dissection in an elderly patient: a case report

**DOI:** 10.4076/1757-1626-2-6645

**Published:** 2009-08-19

**Authors:** Cevher Ozcan, Brian Cambi, Michael Remetz

**Affiliations:** 1 Section of Cardiovascular Medicine, Yale University School of Medicine, 333 Cedar Street, 3 FMP, P.O. Box 208017, New Haven, CT 06520, USA

## Abstract

**Introduction:**

Spontaneous coronary artery dissection is an uncommon cause of acute coronary syndrome and sudden cardiac death. We report an unusual case of multi vessel spontaneous coronary artery dissection in an elderly woman with successful medical management.

**Case presentation:**

A 65 year-old woman with hypertension and Parkinson's disease presented with sudden onset severe chest pain. Electrocardiogram showed ischemic ST-segment elevation in inferior and lateral leads. Urgent cardiac catheterization revealed focal dissections in four small caliber coronary arteries in right and left coronary systems including right posterior decending, posterolateral, obtuse marginal and septal arteries. Angiography demonstrated no significant atherosclerotic coronary artery disease. The patient was medically managed with Eptifibatide, Aspirin, Clopidogrel and β blocker since dissected vessels were not technically suitable for percutaneous coronary intervention. Antiparkinson medications were held as a potential cause of dissection. She responded well to medical management.

**Conclusion:**

Coronary dissection should be considered in all patients with an acute coronary syndrome. Medical management is an effective therapeutic option for the patient with small vessel dissections.

## Introduction

Spontaneous coronary artery dissection (SCAD) is a rare clinical condition that causes acute coronary syndrome (ACS) and sudden cardiac death. It occurs in young or middle aged otherwise healthy patients [1,2]. In fact there has been no reported case of ACS as a result of SCAD in an elderly patient with otherwise normal coronary anatomy. It is often diagnosed post-mortem since the risk of mortality is high in patients with SCAD [1-4]. Here, we present a unique case of multi vessel SCAD in an elderly woman who presented with ACS and survived with medical management.

## Case presentation

A 65-year-old Caucasian woman with history of hypertension and Parkinson's disease presented to an emergency department because of severe sudden onset sub-sternal chest pain radiating to left arm and jaw that woke her up in the morning. Her blood pressure was well controlled with Lisinopril. She was taking multiple anti-Parkinson medications including Levodopa-Carbidopa and Trihexyphenidyl with a recent addition of Rasagiline and Pramipexole. Two months prior to her current illness she was admitted to the hospital with a non ST elevated myocardial infarction and her coronary angiography demonstrated a right dominant circulation without significant coronary artery disease. Echocardiogram revealed normal cardiac structure and function.

**Figure 1:**
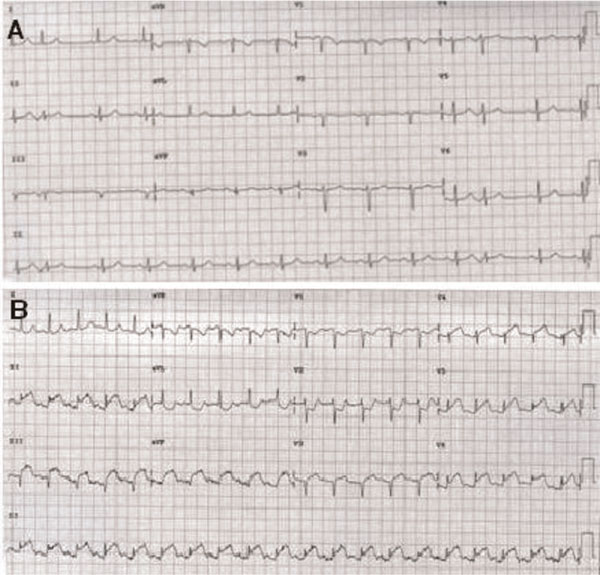
**Electrocardiography showed ischemic ST segment elevation in inferior and lateral leads with reciprocal changes (A), which improved following treatment with thrombolytic (B)**.

At this presentation, her electrocardiogram showed ischemic ST-segment elevation in inferior and lateral leads with reciprocal changes (Figure 1A) while she was haemodynamically stable with an unremarkable physical examination. Initial laboratory tests including complete blood count, electrolytes, renal and liver functions were within normal limits. Later her cardiac enzymes were elevated with peak troponin of 12.2 ng/mL (reference range, <0.04 ng/mL) and creatinine kinase MB fraction of 47 ng/mL (reference range <5 ng/mL).

The medical treatment with Tenecteplase, Enoxaparin, Aspirin, and Metoprolol was started immediately. Shortly after thrombolysis her ST-segments improved (Figure 1B) but she developed ventricular fibrillation and therefore cardiopulmonary resuscitation was performed. Following successful resuscitation, the patient was transferred to our facility for further management.

**Figure 2:**
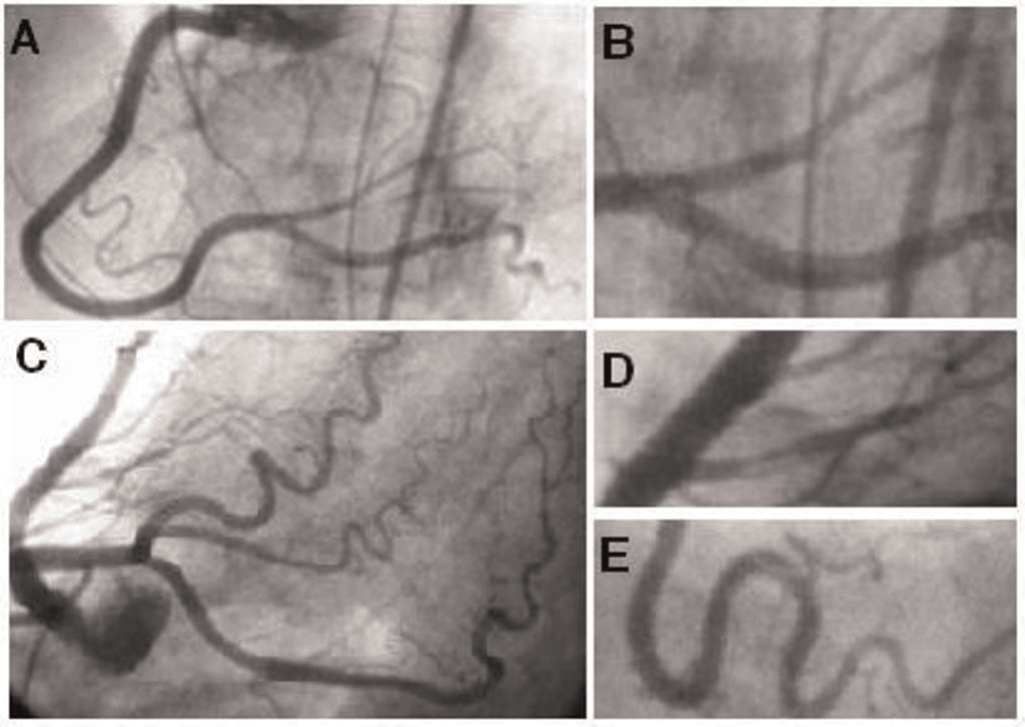
**Coronary angiogram revealed focal spontaneous type C dissections in her right posterior decending artery and a branch of posterolateral artery (A & B), a septal artery (C & D) and an obtuse marginal (C & E)**.

Upon arrival to our cardiac catheterization laboratory, we performed first thoracic aortography that showed normal aortogram with no aortic dissection. Then, coronary angiography was proceeded which revealed focal spontaneous type C dissections in her four coronary artery braches including right posterior descending artery with a branch of posterolateral artery (Figure 2A &2B), an obtuse marginal, and a septal artery (Figure 2C, D &2E). All four vessels with dissections were small in caliber and not technically suitable for percutaneous coronary intervention. Medical management was initiated with Eptifibatide, Aspirin, Clopidogrel and β blocker in addition to holding her medications for treatment of Parkinson's disease. She recovered well with medical management and was discharged home in stable condition.

In order to determine possible reported causes of coronary dissection, other laboratory studies were completed including an erythrocyte sedimentation rate, c-reactive protein, antinuclear antibody and rheumatoid factor levels as well as a toxicology screen. All were negative.

## Discussion

Since first described in 1931, there are fewer than 200 published cases of SCAD [1,2]. Usually they involve the left anterior decending artery in women and right coronary artery in men [2]. Although the etiology and mechanism of SCAD remain unknown, it has been reported to be associated with various clinical conditions including pregnancy, connective tissue disease, blunt chest trauma, physical exercise, systemic lupus, sarcoidosis, vasculitis and medications (oral contraceptives and cyclosporin) [2-4]. Hemodynamic factors, particularly sheer stress, arterial wall changes, proteases released from eosinophils, and intimal tears are hypothesized to cause SCAD [2-4]. Hemorrhage into the media of the coronary artery or between the media and external elastic lamina is main pathogenesis in dissection that results in compression of the true vessel lumen [2-4]. Rupture of a plaque or vasa vasorum in a developing atheroma may lead to intramedial hemorrhage and subsequent dissection. Typically there is angiographic evidence of flow in two lumens, true and false lumens, separated by a radiolucent flap of arterial intima.

The majority of SCAD cases occur in young women with a mean age of 35 to 40 and present with sudden cardiac death [1-4]. About half of the patients who survive their initial event will have a recurrent event in the ensuing two months. In fact, our patient sustained a non ST elevated myocardial infarction 2 months prior to this admission raising the possibility of a SCAD as an etiology behind this earlier presentation. It is usually a single-vessel disease, but multi-vessel involvement has also been described as it was in our case [2,3]. The overall prognosis depends on the vessels involved and clinical presentation. Since it is a fatal disease, 70% of the patients with SCAD are diagnosed post-mortem [2,3]. Although the optimal therapy for SCAD is not yet defined, it should be individualized. As in our case, medical management with glycoprotein IIb/IIIa inhibitor, Clopidogrel, Aspirin and beta-blocker can be effective. The use of thrombolytics remains controversial because of the possibility of extension of dissection with hematoma. Our patient received thrombolytic prior to diagnosis of SCAD as part of treatment of ST-segment elevated myocardial infarction.

This is an unusual case of SCAD in many regards including its occurrence in an elderly woman, involvement of multiple small coronary arteries at the same time, and absence of any known predisposing factor. Parkinson's Disease or antiparkinson drugs are not reported risk factors for SCAD. However it is known that Parkinson's disease is associated with sympathetic neuro-circulatory failure and dysautonomias, which may modify coronary flow and the surrounding hemodynamic milieu. Antiparkinson drugs also reduce coronary flow as secondary to decreased cell membrane permeability [5]. Our patient was on multiple anti-Parkinson medications including Levodopa-Carbidopa, Rasagiline, Trihexyphenidyl and Pramipexole at the time of the event. Thus we believe that our patient's SCAD may have been secondary to antiparkinson medications and/or her Parkinson's disease.

## Conclusion

Coronary dissection should be considered in all patients with an ACS in the absence of obstructive epicardial coronary artery disease. Thus early diagnosis and treatment may reduce the morbidity and mortality of SCAD. Medical management is an effective therapeutic option for patient with a small vessel dissection.

## Abbreviations

ACS: acute coronary syndrome; SCAD: spontaneous coronary artery dissection

## Consent

Written informed consent was obtained from the patient for publication of this case report and accompanying images. A copy of the written consent is available for review by the Editor-in-Chief of this journal.

## Competing interests

The authors declare that they have no competing interests.

## Authors' contributions

All authors were involved in the patient's management including diagnosis and treatment as well as performing cardiac catheterization. CO drafted the manuscript. BC and MR revised it critically for important intellectual content. There is no grant support for this manuscript.
